# Long-term repeatability of peripapillary optical coherence tomography angiography measurements in healthy eyes

**DOI:** 10.1038/s41598-021-03469-4

**Published:** 2021-12-13

**Authors:** Woo Hyuk Lee, Min-Woo Lee, Min-Su Kim, Cheon Kuk Ryu, Jung-Yeul Kim

**Affiliations:** 1 Department of Ophthalmology, Gyeongsang National University Changwon Hospital, Changwon, Republic of Korea; 2 Department of Ophthalmology, Konyang University College of Medicine, Daejeon, Republic of Korea; 3 Department of Ophthalmology, Chungnam National University College of Medicine, #640 Daesa-dong, Jung-gu, Daejeon, 301-721 Republic of Korea

**Keywords:** Optic nerve diseases, Retinal diseases

## Abstract

This is a prospective observational study to establish the short- and long-term repeatability of measurements of peripapillary optical coherence tomography angiography (OCTA) parameters in healthy eyes and identify factors affecting long-term repeatability. We enrolled 84 healthy eyes. Participants with a history of any ophthalmic disease (except high myopia) or intraocular surgery were excluded from the study. An experienced examiner performed OCTA using disc-centered 6 × 6 mm scans. All examinations were conducted twice at 5-min intervals at the initial visit and repeated at least 6 months later. For short-term repeatability, the coefficient of variation (CV) was 2.94–4.22% and the intraclass correlation coefficient (ICC) was 0.840–0.934. For long-term repeatability, the CV was 2.73–3.84% and the ICC was 0.737–0.934. Multivariate analyses showed that the axial length (AL) (B = 0.970; p = 0.002) and mean signal strength (SS) (B = − 2.028; p < 0.001) significantly affected long-term repeatability. Measurements of peripapillary OCTA parameters exhibited excellent short-term and good long-term repeatability in healthy individuals. The mean SS and AL affected long-term repeatability and should be considered while interpreting peripapillary OCTA images.

## Introduction

Optical coherence tomography angiography (OCTA) is an advanced, non-invasive diagnostic technique that can visualize retinal blood vessels based on the flow of red blood cells. OCTA provides a detailed view of the three-dimensional microvascular structure, which is difficult to visualize during fluorescein angiography because of the diffusion of dye^1,2^. Various OCTA or customized software can be used to calculate quantitative parameters based on these images, providing useful information for the diagnosis and follow-up of retinal diseases.

The Cirrus HD-OCT 5000 and AngioPlex software (version 10.0; Carl Zeiss Meditec AG, Dublin, CA, USA) quantitatively analyze retinal vessels in the superficial capillary plexus, providing measures of vessel density (VD) and perfusion density (PD)^3^. These OCTA parameters are calculated automatically and are easy to apply in clinical settings. In addition, glaucoma and neuro-ophthalmic diseases can be diagnosed using peripapillary OCTA^4–9^. Because such parameters are useful in clinical practice, establishing OCTA repeatability and reproducibility were essential, and long-term repeatability is significant for disease follow-up. Previously, many studies have reported good short-term repeatability of OCTA (including peripapillary OCTA) measurements in people with normal and diseased eyes^10–12^. In long-term repeatability, previous our study found that macula OCTA had reasonable repeatability in healthy eyes^13^. Furthermore, Lee et al.^14^ reported good long-term reproducibility of peripapillary OCTA measurements in patients with suspicion of glaucoma. However, the long-term repeatability of peripapillary OCTA measurements in healthy eyes has not been studied. Unlike macular OCTA, in a clinical setting, peripapillary OCTA sometimes requires a manual operation such as the gaze position and disc centering when performing the examination. In addition, to avoid analysis around peripapillary atrophy, a wider range of examinations is often performed, too. So, these points may have the potential to affect long-term repeatability. Therefore, in a clinical setting, establishing of prospective peripapillary OCTA repeatability in healthy eyes is crucial.

In this study, we determined the short- and long-term repeatability of peripapillary OCTA measurements in healthy eyes as well as factors affecting the long-term repeatability of measurements.

## Results

### Demographics

We included 84 healthy eyes in this study. The mean age of study participants was 49.26 ± 17.32 years, and the mean interval between measurements was 12.25 ± 6.98 months. The mean spherical equivalent was − 1.32 ± 2.30 diopters, and the mean axial length was 24.56 ± 1.43 mm (7 eyes, 8.3%, were included as high myopia; axial length > 26.5 mm). The mean central macular, peripapillary nerve fiber layer, and ganglion cell-inner plexiform layer thicknesses were 258.24 ± 21.82, 97.23 ± 14.51, and 84.60 ± 7.23 μm, respectively (Table 1).

**Table 1:** Demographics and baseline characteristics of participants.

Number of eyes	84
Age (years, mean ± SD)	49.26 ± 17.32
Sex [male, n (%)]	39 (46.4%)
BCVA (logMAR, mean ± SD)	− 0.01 ± 0.04
Spherical equivalent (diopters, mean ± SD)	− 1.32 ± 2.30
Intraocular pressure (mmHg, mean ± SD)	14.85 ± 2.84
Axial length (mm, mean ± SD)	24.56 ± 1.43
Interval between visits (month, mean ± SD)	12.25 ± 6.98
Mean CMT (μm, mean ± SD)	258.24 ± 21.82
Mean pRNFL thickness (μm, mean ± SD)	97.23 ± 14.51
Mean GC-IPL thickness (μm, mean ± SD)	84.60 ± 7.23

*SD* standard deviation, *BCVA* best-corrected visual acuity, *CMT* central macular thickness, *pRNFL* peripapillary retinal nerve fiber layer, *GC-IPL* ganglion cell-inner plexiform layer.

### Short-term repeatability

**Figure 1:**
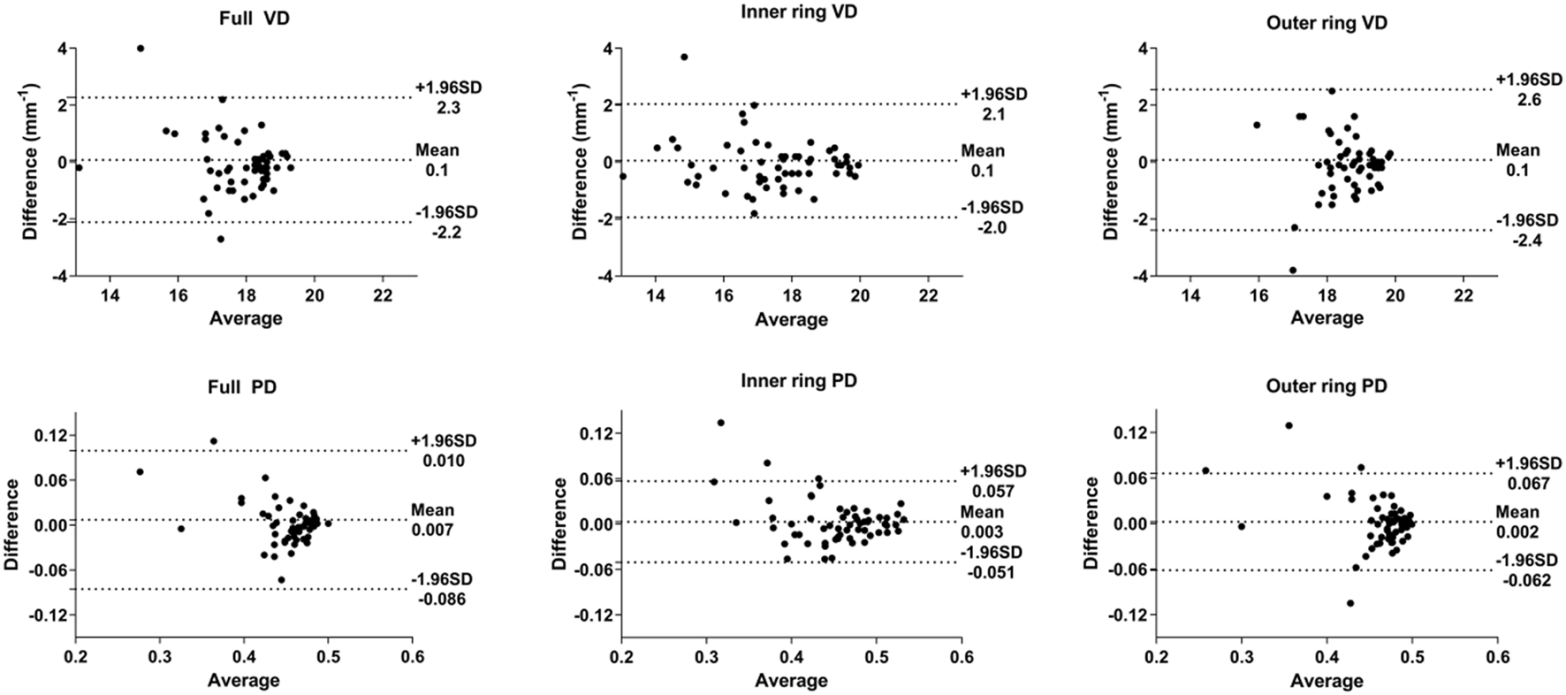
Bland–Altman plots showing the level of agreement for peripapillary optical coherence tomography angiography parameters between two measurements taken at the initial visit. *VD* vessel density, *PD* perfusion density.

Table 2 summarizes the means, ICCs, and CVs for peripapillary OCTA parameters obtained at the initial visit. For VD, the CV was 2.94–4.08% and the ICC was 0.918–0.929, indicating good repeatability for all areas. For PD, the CV was 3.17–4.22% and the ICC was 0.840–0.934, indicating good repeatability. In the Bland–Altman plots, the differences were close to 0 for all parameters (Fig. 1).

**Table 2:** Short-term repeatability of initial measurements of peripapillary optical coherence tomography angiography parameters.

	1st measurement	2nd measurement	ICC	CV (%)
**Vessel density (mm**^**−1**^**)**				
Full	17.59 ± 1.74	17.51 ± 2.36	0.921	3.59
Outer ring	18.19 ± 2.14	18.11 ± 2.72	0.929	4.08
Inner ring	17.28 ± 1.65	17.23 ± 2.02	0.918	2.94
**Perfusion density**				
Full	0.453 ± 0.063	0.446 ± 0.064	0.840	4.08
Outer ring	0.459 ± 0.058	0.457 ± 0.072	0.934	4.22
Inner ring	0.453 ± 0.048	0.450 ± 0.056	0.926	3.17

*ICC* intraclass correlation coefficient, *CV* coefficient of variation.

All values are expressed as the mean ± standard deviation.

### Long-term repeatability

**Figure 2:**
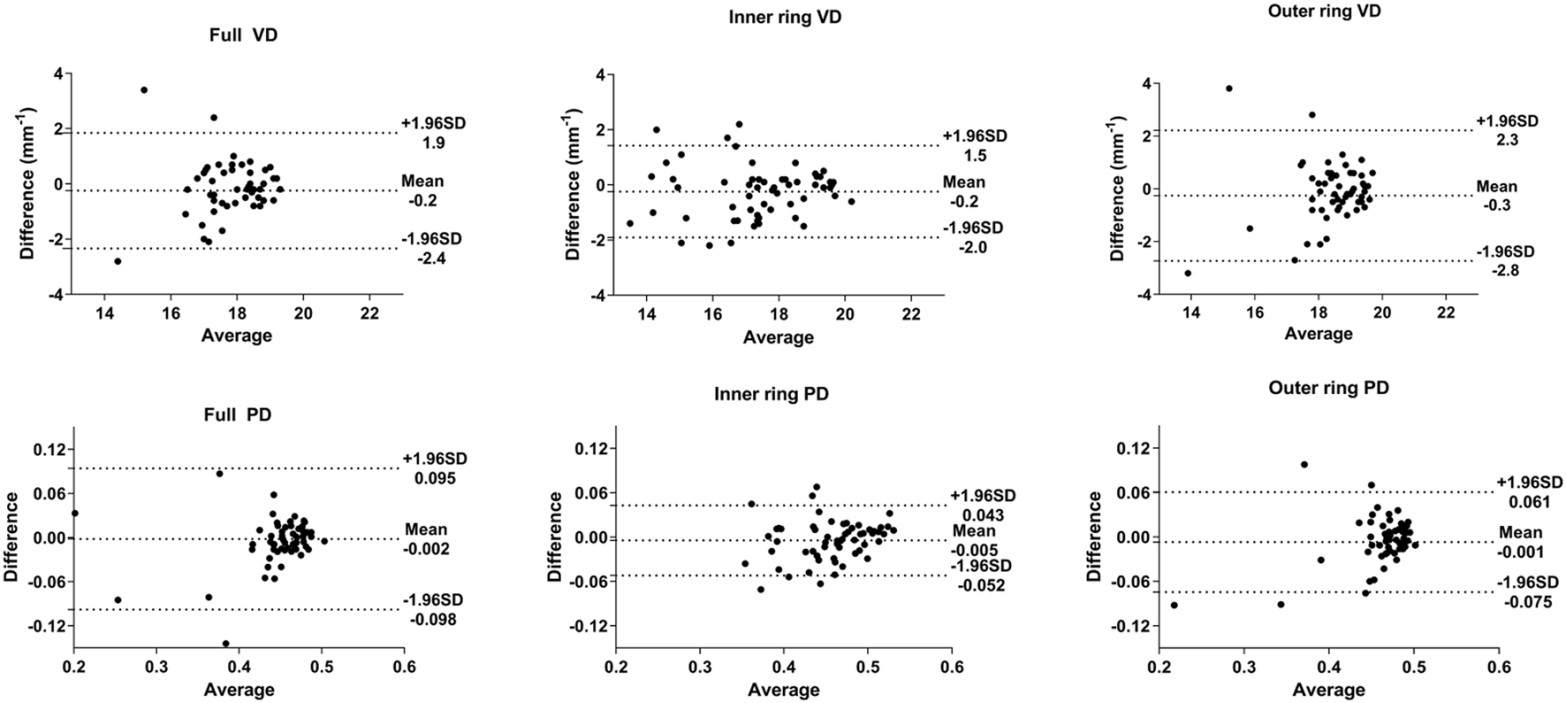
Bland–Altman plots showing the level of agreement for peripapillary optical coherence tomography angiography parameters between two measurements taken at intervals of at least 6 months. *VD* vessel density, *PD* perfusion density.

Table 3 summarizes the means, ICCs, and CVs for peripapillary OCTA parameter measurements obtained at both visits. For VD, the CV was 2.73–3.68% and the ICC was 0.886–0.934. For PD, the CV was 2.99–3.84% and the ICC was 0.737–0.923. In the Bland–Altman plots, the differences were close to 0 for all parameters (Fig. 2).

**Table 3:** Long-term repeatability of initial measurements of peripapillary optical coherence tomography angiography parameters.

	1st measurement	2nd measurement	ICC	CV (%)
**Vessel density (mm**^**−1**^**)**				
Full	17.59 ± 1.74	17.83 ± 1.60	0.886	3.24
Outer ring	18.19 ± 2.14	18.44 ± 1.89	0.891	3.68
Inner ring	17.28 ± 1.65	17.49 ± 1.70	0.934	2.73
**Perfusion density**				
Full	0.453 ± 0.063	0.455 ± 0.042	0.737	3.73
Outer ring	0.459 ± 0.058	0.466 ± 0.050	0.887	3.84
Inner ring	0.453 ± 0.048	0.458 ± 0.043	0.923	2.99

*ICC* intraclass correlation coefficient, *CV* coefficient of variation.

All values are expressed as the mean ± standard deviation.

### Factors affecting the long-term repeatability of OCTA measurements

**Figure 3:**
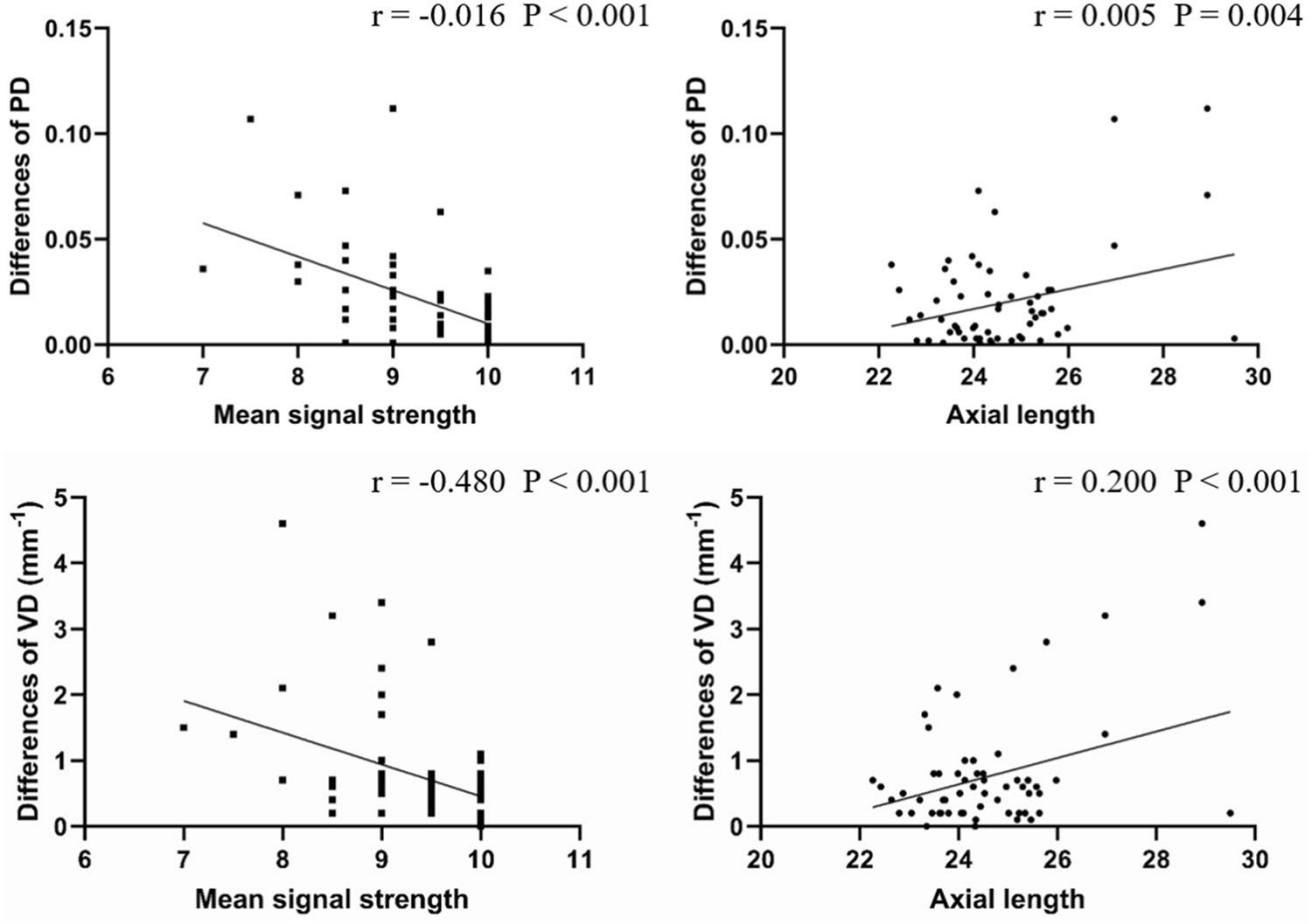
Scatter plots showing the associations of peripapillary optical coherence tomography angiography parameters difference with the mean signal strength (SS) and axial length. Differences of VD and PD were significantly correlated with the mean SS and axial length. Correlation coefficients (r) and p-values are shown. *VD* vessel density, *PD* perfusion density.

Univariate analyses showed that the axial length (B = 1.121; p < 0.001), mean SS of two measurements (B = 2.511; p < 0.001), difference in SS between measurements (B = 1.806; p = 0.001), and mean ganglion cell-inner plexiform layer thickness (B = − 0.127; p = 0.036) affected the long-term measurement repeatability of the parameters (Table 4). Multivariate analyses showed that the axial length (B = 0.970; p = 0.002) and mean SS (B = − 2.028; p < 0.001) affected the long-term measurement repeatability of the parameters. Scatter plots confirmed the associations of the mean SS and axial length with differences of VD and PD (Fig. 3).

**Table 4:** Univariate and multivariate linear regression for the association between clinical and anatomical parameters and coefficient of variation of vessel density.

	Univariate analyses		Multivariate analyses	
	B (95% CI)	P value	B (95% CI)	P value
Age	− 0.052 (− 0.102, − 0.003)	**0.039**	− 0.013 (− 0.062, 0.036)	0.600
Sex	1.367 (− 0.367, 3.102)	0.121		
Laterality	0.001 (− 1.759, 1.761)	0.999		
BCVA	1.260 (− 19.817, 22.336)	0.906		
Spherical equivalent	0.079 (− 0.305, 0.462)	0.684		
Intraocular pressure	− 0.277 (− 0.582, 0.028)	0.074		
Axial length	1.121 (0.554, 1.688)	**< 0.001**	0.970 (0.363, 1.576)	**0.002**
Interval between visits	0.042 (− 0.084, 0.168)	0.506		
Mean SS	− 2.511 (− 3.570, − 1.452)	**< 0.001**	− 2.028 (− 3.137, − 0.918)	**< 0.001**
SS difference	1.806 (0.808, 2.804)	**0.001**	0.872 (− 0.105, 1.850)	0.080
Mean CMT	− 0.017 (− 0.057, 0.024)	0.415		
Mean pRNFL thickness	0.019 (− 0.042, 0.080)	0.535		
Mean GC-IPL thickness	− 0.127 (− 0.246, − 0.009)	**0.036**	0.004 (− 0.104, 0.112)	0.942

*CI* confidence interval, *BCVA* best-corrected visual acuity, *SS* signal strength, *CMT* central macular thickness, *pRNFL* peripapillary retinal nerve fiber layer, *GC-IPL* ganglion cell-inner plexiform layer.

Values with P < 0.05 are shown in bold.

## Discussion

OCTA is a commonly used, advanced technique based on OCT. OCTA allows non-invasive visualization of the vessels and assessment of the optic nerve head (ONH) microcirculation^15^. AngioPlex uses a combination of amplitude- and phase-variance techniques (OMAG) to evaluate the microcirculation and provide quantitative data for clinical use^3^. Jia et al.^15^ were the first to report OCTA results for the macula and ONH using a semi-automated split-spectrum amplitude-decorrelation angiography algorithm. Since then, several studies have investigated differences in the ONH and peripapillary areas between patients with glaucoma or neuro-ophthalmologic diseases and normal eyes^16–18^. Because peripapillary OCTA is used to diagnose glaucoma and other ophthalmic diseases, it is essential to determine the intra- and inter-visit repeatability of peripapillary OCTA in healthy eyes before performing longitudinal studies involving patients with eye diseases. In this study, we investigated the short- and long-term measurement repeatability of peripapillary OCTA parameters in healthy eyes. We demonstrated excellent short-term and good long-term measurement repeatability of these parameters.

In this study, there was good long-term repeatability of peripapillary OCTA parameter measurements, based on a 6 × 6 mm area scan. The CV was 2.73–3.84% and the ICC was 0.737–0.934. Previous studies have also reported good long-term measurement repeatability of peripapillary and ONH OCTA parameters in healthy eyes. Jia et al.^5^ performed ONH OCTA in four normal individuals and glaucoma patients, and reported that the CV representing intra-visit repeatability and inter-visit (1 year) reproducibility of disc flow index measurements was 1.2% and 4.2%, respectively. Chen et al.^19^ reported good long-term repeatability and inter-visit reproducibility within 6 weeks (CV: 1.1–6.8%) for four healthy individuals. However, these studies were not designed to determine the long-term repeatability of measurements. The sample sizes in these studies were small, and the analyses were limited to calculating the CV. Lee et al.^14^ reported good long term reproducibility of peripapillary OCTA measurements in 120 individuals with suspicion of glaucoma, using a clinic-oriented quantification software (Cirrus) (CV 2.02% and ICC 0.852 for PD). Although this study did not include healthy eyes, the results are similar to ours. In healthy eyes study, our previous study with macular OCTA parameters, using a SS > 7 and a 3 × 3 mm area scan, found that OCTA had reasonable long-term repeatability in healthy eyes (CV: 5.39–12.62%)^13^. However, in a clinical setting, peripapillary OCTA sometimes requires a manual control more than macular, such as the gaze position in examination and disc centering when analyzing the results. In addition, a wider scan size is also often needed to avoid the peripapillary atrophy, which may lead to low SS. Considering these factors, we conducted a study with a SS ≥ 7 and a scan range of 6 × 6 mm. However, repeatability was good despite these factors, indicating that peripapillary OCTA can be used to evaluate longitudinal changes.

The mean SS (B = − 2.028; p < 0.001) affected long-term repeatability. Previous studies have also identified the SS as an important factor in OCTA evaluations^11,14,20–23^. Fenner et al.^20^ reported that motion artifacts and TopQ scores (similar to the SS) could be used to evaluate the quality and reliability of OCTA images. Because we excluded images with artifacts, the previous and current studies agree on the importance of SS in the evaluation of OCTA images. Lee et al.^14^ reported that the SS had a significant positive association with the long-term repeatability of OCTA parameter measurements. However, in our previous long-term macular OCTA study, only the differences in SS between two measurements were significant in the multivariate analysis, not the mean SS^13^. This difference in results is probably due to the recruitment of eyes with lower SS (SS ≥ 7). Lei et al.^10^ emphasized improving and maintaining the SS when acquiring OCTA scans because OCTA parameters vary with SS. Therefore, it would be essential to consider both mean SS and SS differences before analyzing OCTA images.

In the present study, the axial length (B = 0.970; p = 0.002) significantly affected long-term repeatability, which means that the longer the axial length, the lower the long-term repeatability. The authors assumed the reason as follows. First, the elongation and stretch of the eyeball create a wider examination range with OCTA in long eyes compared to short eyes. Because a different magnification of OCTA is used to image the retina of a myopic eye^24,25^. Lei et al.^10^ reported that the OCTA scan range influences OCTA parameters because of the image resolution. They found that the 3 × 3 mm scan had a better lateral resolution than the 6 × 6 mm scan. In addition, wide-range scans also affect the centering of scans on the disc during OCTA examination. Therefore, narrower scan ranges are associated with better repeatability and reproducibility. Our previous long-term repeatability study that used smaller scan area (3 × 3 mm) found that the axial length did not affect long-term repeatability^13^. Studies using severely narrow scans reported that the axial length did not significantly influence the VD^12,26^. Li et al.^11^ used criteria similar to those used in our study but different OCTA scan patterns (3 × 3 mm), and found that the axial length had a weakly positive correlation with VD. Second, the elongation and stretch of the eyeball also affected the true vessel size in the myopic eye, even after correction of the magnification effect. Several studies have reported that the axial length was negatively correlated with OCTA parameters in the myopic eye, despite magnification correction^27–30^. Therefore, small capillary sizes may reduce the image resolution and repeatability. In summary, small vessel sizes and a wider scan range reduce the image resolution and OCTA repeatability in the myopic eye. So, we propose that the magnification effect should be adjusted and a narrow OCTA scan should be used to evaluate myopic eyes.

In this study, the long-term repeatability was slightly lower than the short-term. Since the factors affecting the long-term repeatability were mean SS and high myopia. The authors analyzed the mean SS of the two groups (short-term vs. long-term) because the axial length was the same in the two groups. However, the mean SS was not significantly different in the two groups (p = 0.551). Therefore, the authors speculated that the cause was a change in the scan location. The scan location change due to the difference in the gaze position and disc centering may have a more significant difference in the long-term than in the short-term. Especially, high myopia may have a more significant effect on long-term repeatability due to small vessel size and low resolution. However, in this study, the proportion of patients with high myopia was not high for statistical analysis (8.3%), and only one OCTA device was used. So, further research is needed with multiple devices in high myopia.

This study had some limitations. First, we did not exclude participants with high myopia because of the high prevalence of myopia in East Asia^31^. This allowed us to investigate the effects of axial length on peripapillary OCTA repeatability in a real-world population. In addition, we did not correct the effect of magnification due to elongation of the axial length. Future studies should correct the magnification effect while evaluating OCTA repeatability. Third, we only studied the superficial capillary plexus layer because this was the only area for which automated measurements were available. Therefore, studies on the choroid or outer retina may reveal different results from those of this study. However, analysis of the superficial layer is more precise than that of the deep layer because projection artifacts are not seen^1,2^. Lastly, this study was conducted with only one specific OCTA device (Cirrus HD-OCT 5000). Therefore, results may vary in other OCTA devices.

The strength of our study is that this was the first study to prospectively evaluate the long-term repeatability of peripapillary OCTA parameter measurements based on a large sample of healthy eyes. In addition, we also identified the factors affecting the long-term repeatability of peripapillary OCTA parameter measurements.

In conclusion, peripapillary OCTA parameter measurements exhibited excellent short-term repeatability and good long-term repeatability in healthy eyes. Additionally, the mean SS and axial length affected the long-term measurement repeatability of OCTA parameters. Therefore, physicians should consider the SS and axial length while evaluating peripapillary OCTA parameters.

## Methods

### Participants

This prospective, longitudinal observational study was conducted in accordance with the Declaration of Helsinki and approved by the Institutional Review Board and Ethics Committee of Chungnam National University Hospital, Daejeon, Republic of Korea. Written informed consent was obtained from all participants. We enrolled healthy eyes from the Repeatability Study of Optical Coherence Tomography Angiography, a prospective study including healthy eyes and eyes with various diseases treated at the retinal clinic of Chungnam National University Hospital.

All patients underwent ophthalmic assessment at the first and second visits. The ophthalmic assessment included evaluations of the best-corrected visual acuity (BCVA), spherical equivalent, intraocular pressure (IOP), and axial length (IOL Master; version 5.02; Carl Zeiss, Jena, Germany); a fundus examination; spectral-domain optical coherence tomography (OCT); and OCTA. The second visit was scheduled at least 6 months after the first visit.

Participants with a history of any ophthalmic diseases (except high myopia), intraocular surgery (except uncomplicated cataract extraction), a BCVA < 20/20, an IOP > 21 mmHg, or age > 80 years or < 20 years were excluded from the study. Eyes with any abnormal findings upon spectral-domain OCT performed by the retinal specialist (J.Y.K) were also excluded. We additionally excluded myopic eyes with maculopathy more severe than category 1 (tessellated fundus only) or additional lesions (“plus” lesion)^32^. Eyes with floaters (Weiss’s ring) or a membrane anterior to the disc were excluded from the study as well.

### OCTA imaging protocol

**Figure 4:**
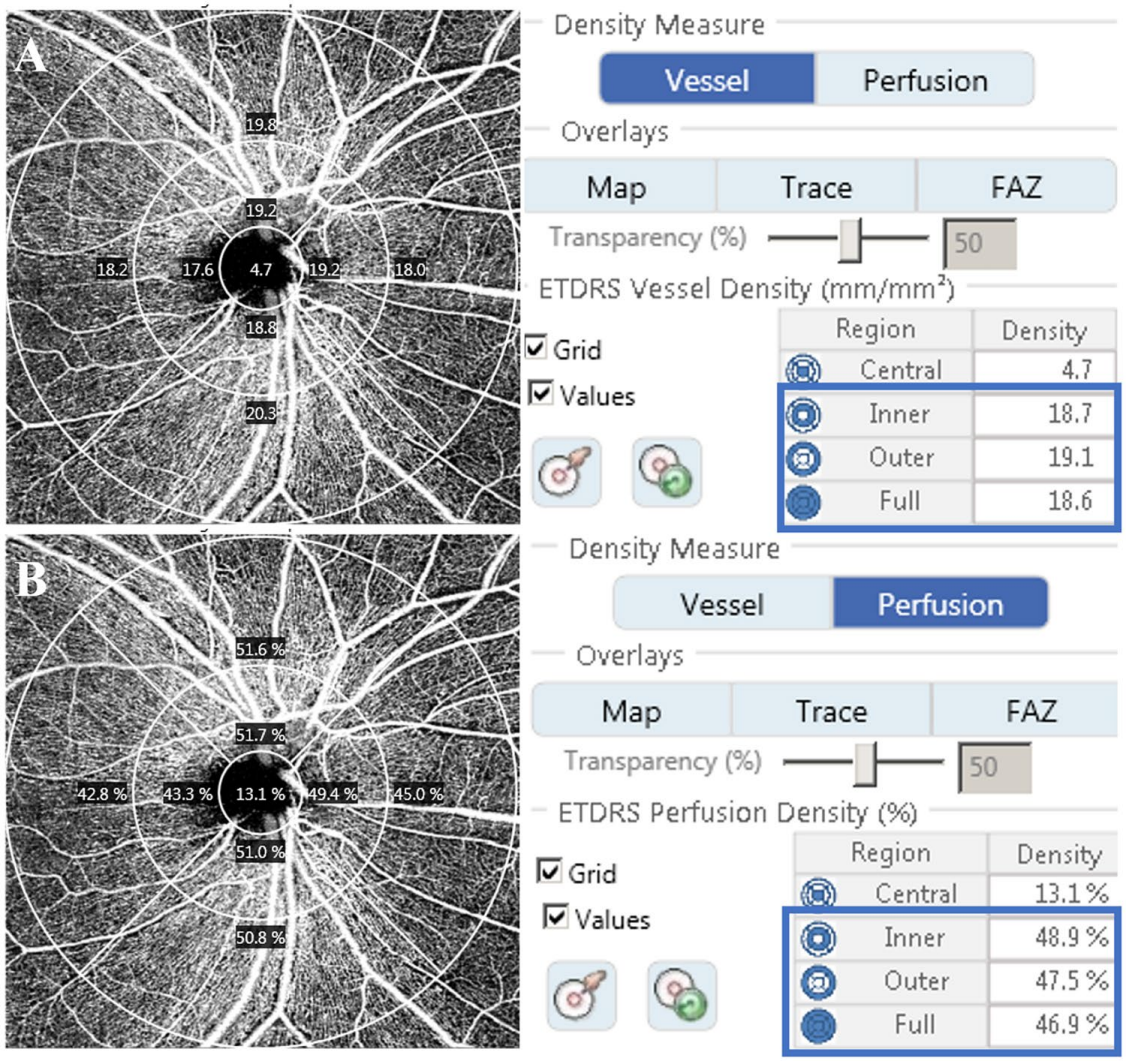
Optical coherence tomography angiography 6 × 6 mm scan centered on the optic disc. En face image of the superficial layer overlain with an Early Treatment of Diabetic Retinopathy Study grid. The diameters of the three concentric circles are 1, 3, and 6 mm, respectively. AngioPlex software measured the (**A**) peripapillary vessel density and (**B**) perfusion density in individual subfields. The bold box shows the average automatic quantitative measurements for the entire area, outer ring, and inner ring.

OCTA images were assessed by a skilled examiner using the Cirrus HD-OCT 5000, AngioPlex software, optical microangiography (OMAG) algorithm, and retinal tracking technology. We measured an area of 6 × 6 mm centered on the optic disc and used the OMAG algorithm to analyze the scans. This 6 × 6 mm scan was divided into a 1-mm center, four-quadrant inner sectors, and four-quadrant outer sectors, identical to the inner circles of the Early Treatment of Diabetic Retinopathy Study (Fig. 4). The scan included a 6-mm circle; the inner and outer areas were calculated as the sum of the four inner and outer quadrants, respectively. VD (total length of perfused vasculature per unit area) and PD (total area of perfused vasculature per unit area) were measured automatically based on blood circulation in the superficial capillary plexus, using the software described previously. The short-term repeatability of these parameters was determined during the initial visit by comparing the values obtained from taking these measurements twice at 5-min intervals.

Images of low quality (loss of fixations, segmentation errors, motion artifacts, and signal strength [SS] < 7) were excluded from this study.

### Statistical analyses

To determine the measurement repeatability of the OCTA parameters, we calculated the intraclass correlation coefficient (ICC) and coefficient of variation (CV). An ICC (ratio of the subject variance to the total variance) close to 1 indicated low variance between the two examinations (poor: ICC ≤ 0.40; fair: 0.40 < ICC ≤ 0.59; good: 0.60 < ICC ≤ 0.74; excellent: 0.75 < ICC ≤ 1.00). The CV (%) was calculated as standard deviation/overall mean × 100, with a CV < 10% indicating good repeatability. Bland–Altman plots were used for determining the agreement between two measurements. To evaluate the factors affecting the long-term repeatability of OCTA parameters, linear regression analyses were performed to assess the CV of VD using demographic and ocular variables. Multivariate analyses were performed to assess factors that were observed to affect long-term repeatability on linear regression analysis. Statistical analyses were performed using SPSS Statistics software (version 23.0; IBM Corp., Armonk, NY, USA).

## Data Availability

Data supporting the findings of the current study are available from the corresponding author on reasonable request.
